# Adipocytes: active facilitators in epithelial ovarian cancer progression?

**DOI:** 10.1186/s13048-020-00718-4

**Published:** 2020-09-23

**Authors:** Lan Dai, Keqi Song, Wen Di

**Affiliations:** 1 Department of Obstetrics and Gynecology, Ren Ji Hospital, School of Medicine, Shanghai Jiao Tong University, Shanghai, 200127 China; 2 Shanghai Key Laboratory of Gynecologic Oncology, Ren Ji Hospital, School of Medicine, Shanghai Jiao Tong University, Shanghai, 200127 China; 3 State Key Laboratory of Oncogene and Related Genes, Shanghai Cancer Institute, Ren Ji Hospital, School of Medicine, Shanghai Jiao Tong University, Shanghai, 200127 China

**Keywords:** Epithelial ovarian cancer, Adipocytes, Cancer progression

## Abstract

There is growing evidence that adipocytes play important roles in the progression of multiple cancers. Moreover, in obesity, adipocytes alter their original functions and contribute to the metabolic and inflammatory changes of adipose tissue microenvironment, which can further enhance tumor development. At present, the roles of adipocytes in the pathogenesis of epithelial ovarian cancer (EOC) are far from being fully elucidated. Herein, we summarized the recent advances in understanding the roles of adipocytes in EOC progression. Adipocytes, close neighbors of EOC tissue, promote EOC growth, invasion, metastasis and angiogenesis through adipokine secretion, metabolic remodeling and immune microenvironment modulation. Moreover, adipocytes are important therapeutic targets and may work as useful anticancer drug delivery depot for EOC treatment. Furthermore, adipocytes also act as a therapeutic obstacle for their involvement in EOC treatment resistance. Hence, better characterization of the adipocytes in EOC microenvironment and the crosstalk between adipocytes and EOC cells may provide insights into EOC progression and suggest novel therapeutic opportunities.

## Introduction

Adipose tissue, the main part of human body, is found mainly under the skin but also in deposits such as muscles, intestines, omentum and bone marrow. For a long time, adipocytes, the major components of adipose tissue, were considered as simple depots to store and provide energy. However, since the discovery of adipocyte hormone leptin in 1994 [1], more than 400 adipocyte-secreted factors have been found and adipocytes have now also been regarded as a main source of various endocrine and paracrine factors [2]. These adipocyte secreted products, known as “adipokines” (also called adipocytokines), include hormones (e.g. leptin, adiponectin and resistin), inflammatory cytokines [e.g. tumor necrosis factor-α (TNF-α), interleukin (IL)-6 and IL-8], enzymes [e.g. 17β-hydroxysteroid dehydrogenase (17βHSD) and 11βHSD1], and other factors [3]. Many adipokines, such as leptin, IL-6 and IL-8, have been found to promote the growth, metastasis and drug resistance of different types of tumor [4, 5]. Accordingly, it is tempting to speculate that adipose tissue microenvironment (ATME) may be favorable for tumor progression.

Obesity, a pathological condition accompanied by an excessive growth of adipose tissue, is increasing worldwide, and there is growing evidence for a link between obesity and cancer [6, 7]. This association is partly driven by ATME evolution induced by obesity. During weight gain, adipocytes accumulate lipids, become hypertrophic and eventually die. Adipocyte death triggers immune response and causes aggregation of immune cells [8]. This phenomenon could alter the adipokine profile in ATME with reduced adiponection and increased factors such as leptin, TNF-α and IL-6 [9]. Moreover, obesity-induced adipokine profile change could lead to the metabolic and inflammatory alteration of ATME [9], which further promotes tumor development and contributes to worse cancer prognosis [4, 10]. Therefore, obesity, which causes the dysfunctional state of adipocytes, was more prone to provide a favorable environment for tumor progression.

Epithelial Ovarian cancer (EOC) is the most lethal gynecological malignancy. About 70% of patients are found to have advanced tumors at the time of initial diagnosis, with the disease spread beyond the primary site. This leads to a high mortality rate for EOC. Therefore, an understanding of the mechanisms that regulate the motility and invasive behavior of EOC may have crucial impact on the outcomes of this deadly disease. Clinical observation and retrospective clinical studies suggest that epithelial ovarian carcinomas rarely metastasize outside the adipocyte-rich environment of the peritoneal cavity [11]. Moreover, EOC has a clear predilection for metastasis to the adipose tissues within the abdominal cavity, such as omentum [12–14]. Furthermore, Adipocyte-rich niche is rich in nutrients and growth factors for EOC growth [15]. Thus, it is reasonable to infer that EOC may be particularly affected by the adipocyte-rich microenvironment. However, the mechanisms underlying EOC and adipocyte relationship are not well understood.

In this review, we will focus on the roles of adipocytes on EOC growth and metastasis, and discuss the possibility of adipocytes as novel targets for ovarian cancer therapy.

## Adipocytes and EOC cells: close neighbors

EOC is composed of a diverse group of tumors which can be divided into two categories. The first category, designated type I, is composed of low-grade serous, low-grade endometrioid, clear cell, mucinous and transitional (Brenner) carcinomas. The second category, designated type II, is composed of high-grade serous ovarian cancer (HGSOC), undifferentiated carcinoma and malignant mixed mesodermal tumors. The most common histological subtype is serous ovarian cancer, which may arise from fallopian tube epithelium that implants on the ovary. Endometrioid and clear cell tumors may arise from endometriosis. Preliminary data suggest that mucinous and transitional tumors may arise from transitional epithelial nests [16, 17].

The most common site of EOC metastasis is the omentum. About 80% of the patients with serous ovarian cancer, the most frequent subtype of EOC, present with omental metastasis. The great omentum, a large (about 20 × 12 × 3 cm) fatty pad, covers the majority of the abdominal organs, just like an “apron” [11]. As omentum is rich in adipocytes, EOC cells are in the vicinity of adipocytes during tumor growth and progression. The close contact between EOC cells and adipocytes leads to profound phenotypic alterations of adipocytes. EOC metastasis to the omentum can reduce the size and number of adipocytes at the invasive front compared with adipocytes elsewhere [15]. Adipocyte-EOC cell co-culture could lead to increased fatty acids production of adipocytes [15]. These adipocytes, altered by metastatic cancer cells to obtain the characteristics different from those of primary adipocytes, were named as cancer associated adipocytes (CAAs) [18, 19]. The mechanisms of the functional and phenotypic modifications in EOC associated adipocytes need to be characterized in the future. Furthermore, upon prolonged exposure to EOC, adipocytes in the omentum may lose their lipids content completely and disappear [15], fibroblast-like cells accumulate, which are thought to further strengthen EOC progression [20, 21].

## Role of adipocytes in EOC progression

The growth and metastasis of EOC are closely related to the surrounding microenvironment. The cells, such as adipocyte, in the tumor microenvironment contribute to the metastasis, growth and angiogenesis of ovarian cancer.

### Adipocyte-induced metastasis

**Fig. 1:**
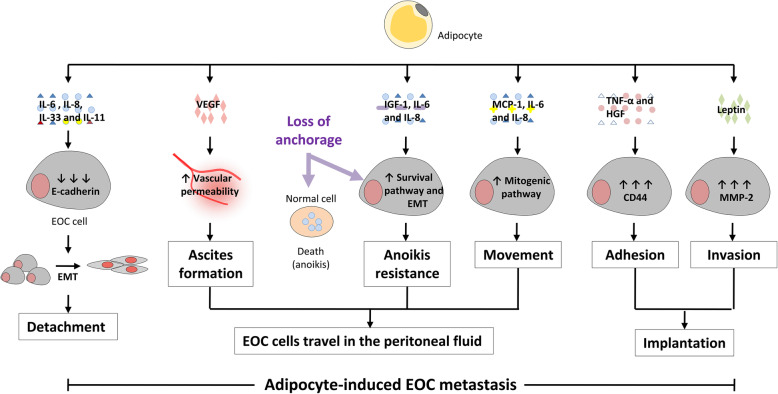
The role of adipocytes in EOC metastasis. Adipocyte-secreted cytokines, such as IL-6, IL-8, IL-11 and IL-33, induced EMT and detachment of EOC cells by inhibiting E-cadherin expression. Adipocytes affect the travel of EOC cells in the peritoneal fluid through modulating several functions including (1) ascites formation by secreting VEGF, which increases vascular permeability; (2) EOC anoikis resistance by secreting IGF-1, IL-6 and IL-8, which activate the survival pathway and EMT of EOC; (3) EOC movement by secreting MCP-1, IL-6 and IL-8, which activate the mitogenic pathway of EOC. Adipocytes affect EOC implantation through modulating (1) EOC adhesion by secreting TNF-α and HGF, which promote CD44 expression; (2) EOC invasion by secreting Leptin, which promote MMP2 expression. Together, these roles of adipocytes facilitate adipocyte-induced EOC metastasis

Unlike the well-studied and classic pattern of hematogenous metastasis found in most other cancers, EOC has a unique way of dissemination [11]. Direct intraperitoneal seeding is the most common route of dissemination of EOC cells to the peritoneum and omentum. The general process of intraperitoneal seeding can be summarized as follows: (1) EOC cells detach from the primary tumor; (2) EOC cells travel within the peritoneal fluid; (3) EOC cell implantation. Adipocytes can influence various steps of the above process (Fig. 1).

#### Adipocytes and EOC cell detachment

The first step for EOC metastasis is leaving the primary tumor. Before EOC cells detach, they often undergo epithelial mesenchymal transition (EMT), which decreases the adhesion between tumor cells. An important molecular crucial for EMT is E-cadherin, a membrane glycoprotein located at cell adhesion junctions [22, 23] and anchored epithelial cells to each other. In epithelial cancer, E-cadherin loss is related with EMT and invasive phenotype acquisition [24]. In EOC, the E-cadherin expression level of cancer cells in ascites or in matastatic lesions is lower than that in the primary tumor [25]. EOC cells with low E-cadherin expression level are more invasive [25, 26]. Moreover, negative E-cadherin expression in EOC can predict a poor patient survival [27]. E-cadherin is notably controlled by zinc-finger transcriptional repressors, such as Snail, Slug, zinc finger E-box binding homeobox 1 (ZEB1) and Twist [23, 28]. Adipocytes may support the detachment of EOC cells by secreting a series of cytokines. For example, IL-8 is secreted by adipocytes and could act locally or systemically [29, 30]. The IL-8 level is increased in both serum and ascites of ovarian cancer patients [31, 32]. Previous study showed IL-8 induced EMT in human EOC cells by inhibiting E-cadherin [33]. Moreover, IL-8 was reported to activate Akt/Slug pathway, which induced the suppression of E-cadherin [34]. IL-6 is one of the most abundantly secreted cytokines of omental adipocytes [15] and is enriched in the malignant ascites from ovarian cancer patients [35]. IL-6 was reported to induce EMT by activating signal transducer and activator of transcription 3 (STAT3) [36], which could induce the down-regulation of E-cadherin [37]. IL-11, an IL-6 family cytokine, can also be secreted by adipocytes [38] and its receptor is commonly expressed in EOC [39]. IL-11 was reported to activate STAT3 [40], a known regulator of E-cadherin and EMT [37]. IL-33, a recently identified IL-1 gene family member, could be secreted by adipocytes [41] and is particularly highly expressed in EOC metastatic tumors [42]. IL-33 was reported to induce the invasive potential of EOC by activating extracellular regulated protein kinases (ERK) pathway [42], an important regulator of E-cadherin and EMT [43].

#### Adipocytes and EOC cell within the peritoneal fluid

After EOC cells detach from the primary tumor, they float within the peritoneal fluid, which carry them to the peritoneal surface and omentum through physiological movement. Adipocytes influence not only the formation of ascites but also the survival and movement of EOC cells in ascites.

##### Adipocytes and ascites formation

Ascites play important roles in carrying EOC cells to distant metastasis sites. Many factors contribute to ascites formation in EOC. One of the cytokines important for ascites formation is vascular endothelial growth factor (VEGF), which promotes the production of ascites through increasing vascular permeability [44]. Moreover, VEGF blockage could significantly inhibit ascites formation in xenograft ovarian cancer mouse models [45]. On one hand, adipocytes can secrete VEGF, which was reported to be regulated by insulin or hypoxia and be associated with adipose tissue accretion [46, 47]. In the rat, VEGF secretion and concentration is depot dependent and highest in omentum [47]. On the other hand, adipocytes may promote the VEGF secretion of EOC cells. IL-6, secreted by adipocytes and accumulated in ascites, was reported to be able to promote VEGF secretion through activating STAT3 pathway [48].

##### Adipocytes and EOC cell survival in ascites

Usually, the cells of an organ only grow and differentiate in the correct context within the tissue. Cells sense their location through specific communication with the surrounding extracellular matrix (ECM) and neighboring cells. When cells detach from the correct context, anoikis, a form of anchorage-dependent cell death, will occur to prevent ectopic cell growth [49, 50]. In other words, anoikis is apoptosis induced by inappropriate cell-context interactions. Therefore, resistance to anoikis is an essential prerequisite for the detached EOC cells to survive in the ascites before arriving at the metastatic sites.

EOC cells can prevent anoikis by activating survival signals, due to paracrine stimulation of neighboring adipocytes. For instance, Insulin-like growth factor 1 (IGF-1), secreted by adipocytes and preadipocytes [51], activates serine/threonine protein kinase (AKT) and ERK pathways, the important survival signals associated with anoikis resistance of EOC [52]. IL-6, secreted by adipocytes, activates STAT3 pathway, another signal related to anoikis resistance [53].

Another primary strategy for EOC to avoid anoikis is EMT activation. E-cadherin loss is an important feature of EMT. Increasing evidence shows that loss of E-cadherin plays crucial roles in anoikis resistance and efficient tumor metastasis [54, 55]. Adipocytes promote EMT of EOC cells through inhibiting E-cadherin expression, as we discussed earlier, thus contributing to anoikis resistance of EOC.

##### Adipocytes and EOC cell movement in ascites

It is thought EOC may metastasize to the peritoneum or omentum through passive mechanisms, induced by physiological movement of the ascites. If metastasis is a random event, all organs in contact with ascites should have the same chance of metastasis. However, both primary and recurrent EOC tend to metastasize to adipose tissue. Adipocytes could attract EOC cells to move to them. In vivo, when HGSOC cells were injected into nude mice abdominal cavity to mimic the intraperitoneal metastasis of EOC, the vast majority of HGSOC cells would move to the omentum, an organ primarily composed of adipocytes [15]. In vitro, both human omental adipocytes and adipocyte-conditioned medium could induce the migration of HGSOC cells [15]. It was also confirmed that adipocytes promoted EOC cell migration through secreted a series of cytokines, such as IL-6, IL-8, monocyte chemoattractant protein-1 (MCP-1) and tissue inhibitors of matrix metalloproteinases (TIMP)-1 [15].

#### Adipocytes and EOC cell implantation

The binding of EOC cells to the mesothelial cells is the first step of EOC implantation [11]. cluster of differentiation (CD)44, the principal cell surface receptor for hyaluronic acid, is an important mediator of EOC cell adhesion [56]. Inhibition of CD44 had been reported to limit intra-abdominal spread of ovarian cancer cell in nude mice [57]. TNF-α, secreted by adipocytes [58], differentially modulates the expression of CD44 in ovarian cancer cells. TNF-α increased CD44 expression in EOC cells by activating c-Jun N-terminal kinase (JNK) pathway. On the contrary, if JNK activation failed to be induced, both CD44 expression and the adhesion ability of ovarian cancer would be inhibited [59]. Hepatocyte growth factor (HGF) is also secreted by adipocytes [60] and its receptor is over expressed by EOC. HGF was reported to enhance the adhesion ability of breast cancer cells through up-regulation of CD44 [61]. Whether HGF can induce CD44 expression in ovarian cancer cells needs further study.

When EOC cells bind to mesothelial cells, they will degrade the extracellular matrix structures to promote implantation. Matrix metallopeptidase (MMP)-2, produced by EOC cells, is a crucial molecular in this step. In vivo, MMP-2 inhibition before EOC cell adhesion significantly reduced the metastasis number and metastasis size in a mouse model of ovarian cancer [62]. Leptin, secreted by adipocytes, has been shown to promote MMP2 production and cell invasion in different kind of cell lines [63, 64]. The roles of leptin on EOC invasion needs to be investigated.

### Adipocyte-induced proliferation

Adipose tissue is a major energy storage organ, the main function of which is the storage of triglycerides for future use. Under fasting conditions or times of elevated energy demands, adipocyte lipolysis leads to the breakdown of stored triglycerides to release free fatty acids (FFAs) and glycerol. Moreover, adipose tissue is also a source of various endocrine and paracrine factors [2]. Upon paracrine interactions with EOC cells, adipocytes provide high-energy metabolites and a series of adipokines contributed to the growth of EOC [15].

#### Adipocytes promote the growth of EOC

Adipocytes can significantly promote the proliferation rate of EOC and transfer nutrition to EOC cells. In vitro, co-culture of HGSOC cells with adipocytes led to an increase in HGSOC proliferation. In vivo, subcutaneous injection of HGSOC cells with adipocytes into nude mice produced tumors larger than tumors produced by HGSOC cells alone. Adipocytes were known to transfer nutrition to surrounding cancer cells as energy supply. Indeed, co-culture of HGSOC cells with adipocytes that had been loaded with fluorescently labeled lipids resulted in cytoplasmic fluorescent lipid droplet accumulation in HGSOC cells, which confirmed the transfer of lipids from adipocytes to these cells. Consistent with these results, in cancer tissue from EOC patients, the cancer cells at the adipocyte-cancer cell interface contained abundant lipids [15].

Resistance to mitochondria-initiated apoptosis provides a growth advantage for cancer cells [50]. Adipocytes may provide a growth advantage for EOC by inducing apoptosis resistance. Both anti-apoptotic members [B-cell lymphoma 2 (BCL-2), B-cell lymphoma-extra large (BCL-X_L_)] and proapoptotic members [BCL2-associated X protein (BAX), Bcl-2 interacting mediator of cell death (Bim)] of BCL-2 family play pivotal roles in controlling mitochondria-initiated apoptosis [65]. Adipocytes can regulate the apoptosis resistance of EOC through modulating BCL-2 family members. For instance, IL-6, secreted by adipocytes, could induce apoptosis resistance of EOC partly by increasing the expression level of apoptosis inhibitory protein (BCL-2, BCL-X_L_) [66]. Similarly, IL-8, another adipocyte-secreted cytokine, could also induce apoptosis resistance of EOC partly by regulating the expression of BCL-2 and BCL-X_L_ [67].

#### The metabolic alteration of EOC influenced by adipocytes

Metabolism alterations happen in cancer cells to meet the extreme energy needs for tumor progression [68]. One of the most famous examples of this phenomenon is “Warburg effect”, a significant shift in metabolism wherein most cancer cells rely on aerobic glycolysis to generate energy [69]. The heterogeneity in cancer metabilism is strongly influenced by the tumor microenvironment. In EOC, the adipocyte rich microenvironment can play essential roles in EOC lipid metabolic alteration. Co-culture of HGSOC cells with adipocytes increased the rate of β-oxidation, a lipid metabolism process by which fatty acids are broken down in the mitochondria and/or in peroxisomes to produce energy [15]. This was paralleled by an increase in the phosphorylation of AMP-activated protein kinase (AMPK) and the mRNA levels of carnitine palmitoyltransferase 1 (CPT1). AMPK, a serine-threonine kinase, plays central role in lipid metabolism by inhibiting lipogenesis and activating β-oxidation [70]. CPT1, a downstream protein of AMPK pathway, is the rate-limiting enzyme which transfers long-chain fatty acyl CoA to mitochondria for β-oxidation [71]. Therefore, alteration of lipid metabolism allows EOC to gain energy on lipids from surrounding adipocytes.

#### The metabolic alteration of adipocytes induced by EOC

Metabolic alteration can occur not only in cancer cells but also in tumor adjacent cells. As described in the “reverse Warburg effect”, a metabolic shift to aerobic glycolysis can occur in the breast cancer adjacent cancer-associated fibroblasts (CAFs), which cause lactate production in CAFs [72]. In EOC, tumor cells play essential roles in metabolic alteration of its adjacent adipocytes. Cultured with HGSOC cells, adipocytes can release more FFAs and glycerol than adipocytes cultured alone [15]. This was paralleled by an increase in the phosphorylation of hormone-sensitive lipase (HSL) and the mRNA levels of Perilipin, the rate-limiting enzymes in triglyceride hydrolysis [73] and the lipid droplet gate-keeper [74], respectively. Moreover, β-adrenergic receptor stimulation usually induces lipolytic activation in adipocytes [75]. HSL activation in adipocytes induced by HGSOC cell could be partially reversed by the β-adrenergic receptor antagonist [15]. Therefore, alteration of lipid metabolism allows adipocytes to provide energy to surrounding EOC. The most remarkable feature of cancer associated adipocytes, smaller size and diminished lipid droplets [76], can partly be explained by the altered lipid metabolism, which produced FFAs through lipolysis to support surrounding cancer cells.

### Adipocyte-induced angiogenesis

When tumors reach a certain size, they require new blood vessel formation (angiogenesis) for nutrition supply, a fundamental event in the process of tumor growth and metastasis. The process of angiogenesis is highly complex and dynamic, and regulated by a series of pro- and anti-angiogenic molecules [77, 78]. Adipocytes could help to create a microenvironment favorable for angiogenesis during EOC progression.

Adipocytes were shown to secrete a number of angiogenesis-associated factors, such as VEGF-A, IL-6 and HGF [46, 60, 79]. VEGF-A, the most well-studied angiogenic factor, plays a pivotal role in EOC angiogenesis by stimulating endothelial cells to form new blood vessel and regulating their permeability [80]. Due to the central role of VEGF in EOC angiogenesis, the VEGF pathway becomes a major focus of target in EOC therapy. Bevacizumab, a humanized anti-VEGF monoclonal antibody, has shown promising results in EOC treatment [81]. IL-6, secreted by both adipocytes and EOC cells, is a potent proangiogenic cytokine [82]. In vitro, IL-6 could promote the tube formation ability of endothelial cell lines established from ovary and mesentery of mice [82]. Moreover, IL-6, secreted by adipocytes, was reported to promote resistance to anti-VEGF therapy of some tumors [83]. HGF, well-known adipokine secreted by adipocytes [60], promotes angiogenesis in multiple pathological conditions. In ovarian cancer, HGF could increase the proliferation, migration and tube-like structure formation in microvascular endothelial cells. Moreover, these effects could be blocked by the HGF receptor inhibitor PF04217903 [84].

EOC progression can lead to local hypoxia of surrounding adipose tissue, which drives hypoxia-induced angiogenesis. When EOC metastasize to the adipocyte tissue, it will take abundant energy from adipocytes for further progression. This ‘parasitic’ mode of EOC survival leads to reduction of adipocytes and areas of hypoxia [85], a triggering event of angiogenesis. Adipocyte-specific deletion of hypoxia-regulated genes those are essential in angiogenesis, such as hypoxia inducible factor (HIF)1A and VEGFA, significantly reduced adipocyte tissue vascularity [86, 87].

### Immune microenvironment modulation by adipocytes

**Fig. 2:**
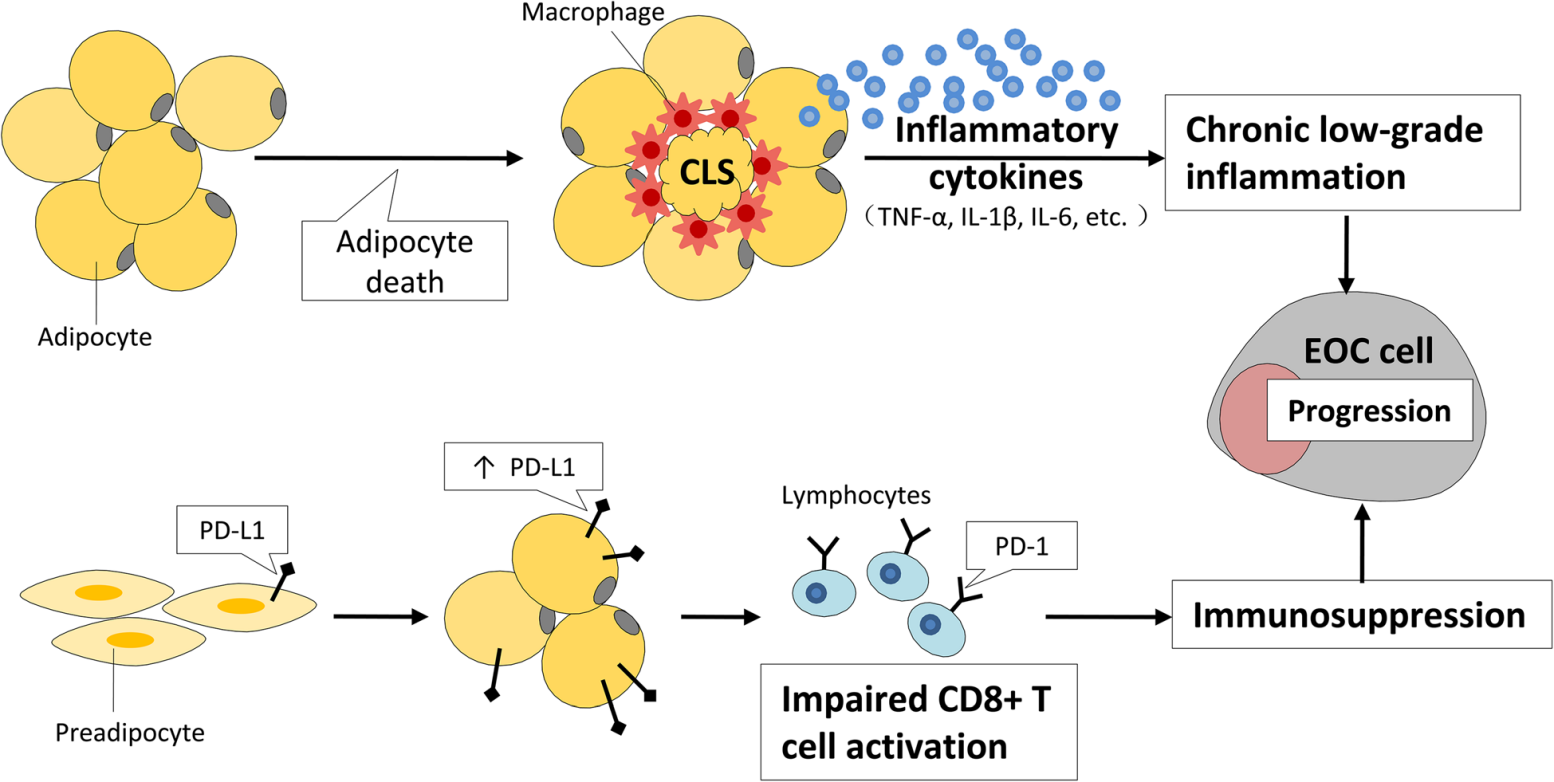
The role of adipocytes in immune microenvironment modulation. Adipocyte death leads to macrophage infiltration into adipose tissue, where they encircle the dying adipocytes to form crown-like structures (CLS). These macrophages are associated with the secretion of multiple inflammatory factors, which modulate the microenvironment to a state of chronic low-grade inflammation. Moreover, increased expression level of PD-L1 in mature adipocytes could interact with T cell surface PD-1 to impair CD8+ T cell activation and cause immunosuppression. Together, these roles of adipocytes facilitate evolution of the immune microenvironment and EOC progression

Aside from having direct effect on EOC progression through paracrine signaling as discussed previously, adipocytes can also have indirect effects on EOC through modulating the immune microenvironment (Fig. 2). Immune microenvironment is believed to be a major factor in EOC initiation and progression. Factors associated with inflammatory responses of ovarian epithelium, such as ovulation and endometriosis, increase the risk of EOC [88, 89]. Moreover, immune microenvironment contributes to increased tumor growth, metastasis and angiogenesis of EOC [90, 91].

#### Chronic low-grade inflammation induced by adipocytes

Adipose tissue consists of a variety of immune cell types (for example, macrophages, T cells and mast cells) that support tissue homeostasis. During weight gain, adipose tissue can modulate the microenvironment to a state of chronic low-grade inflammation [92, 93]. This inflammation has mainly been attributed to areas of adipose tissue where infiltrating macrophages surround the dead or dying adipocytes to form crown-like structures (CLSs) [92, 94, 95]. CLSs, existing in most adipose tissue, show greater frequency in visceral adipose tissue than in subcutaneous tissue [95]. The number and density of CLSs increase with body mass index (BMI) and increased adipocyte size [96]. CLSs are more prevalent in postmenopausal women compared with premenopausal women [97]. Adipocytes could modulate adipose tissue macrophage (ATM) differentiation and function through paracrine role, such as releasing exosomes [98]. ATMs were reported to be associated with the secretion of multiple inflammatory factors, such as TNF-α, IL-1β and IL-6 [92, 93, 99]. These cytokines, contributing to the formation of inflammatory microenvironment, have been shown to stimulate EOC cell growth and influence the clinical disease status and prognosis [82, 100, 101]. Moreover, interaction with macrophages plays key roles in EOC progression. In vitro, co-culture of macrophages with EOC cells could promote cancer cell invasion through activating nuclear factor kappa B (NF-κB) and JNK pathway [102]. In vivo, depletion of peritoneal macrophages reduced ovarian cancer progression by affecting the expression of stromal VEGF [103]. In summary, during weight gain, adipocytes induce a chronic low-grade inflammation microenvironment mainly by modulating infiltrating macrophages. Interactions between EOC and the inflammatory microenvironment increase the growth and metastasis of EOC. Therefore, adipocyte-inflammation-EOC axis is associated with EOC progression.

#### Immunosuppressive effects of adipocytes

Adipocytes play an immunosuppressive role by expressing programmed death-ligand 1 (PD-L1). PD-L1, encoded by the gene CD274, interacts with the corresponding receptor programmed cell death protein 1 (PD-1) on the surface of immune cells, which inhibits the antitumor activity of immune cells and allows cancer cells to escape immune surveillance [104]. PD-L1 expression level in mature adipocytes is significantly higher than that in preadipocytes [105], and has been found to be markedly elevated in brown adipose tissue compared to white adipose tissue [106]. In addition to the PD-L1 expressed on the cell surface of adipocytes, it was also found a sub-population of internally localized endogenous PD-L1 in adipocytes. This internal pool of PD-L1 could influence the antitumor immunity via its active redistribution to the cell membrane [107]. In vitro*,* adipocyte surface PD-L1 could interact with T cell surface PD-1 to weaken the antitumor function of T cell. In vivo, adipocyte PD-L1 could dampen interferin (IFN)γ production of CD8+ T cells. Furthermore, adipogenesis inhibition selectively reduced the PD-L1 expression in adipose tissue and enhanced the antitumor effect of anti-PD-L1 or anti-PD-1 antibodies [106]. These findings suggested the immunosuppressive role of adipose tissue may promote cancer progression. Given the unique adipocyte-rich metastatic niche of ovarian cancer, the immunosuppressive role of adipocytes may explain the low response of EOC to the checkpoint blockade immunotherapies.

## Adipocytes and ovarian cancer treatment

Conventional treatment for EOC includes surgery, chemotherapy and radiotherapy, which largely target tumor cells. With growing interest in immunotherapy, more and more strategies are focused on targeting the immune cells in tumor microenvironment [108, 109]. Targeting tumor microenvironment is becoming an effective strategy to treat cancer [110]. Since adipocytes play important roles in the growth, metastasis and angiogenesis of EOC, therapeutic interventions target or regulate adipocytes might be effective strategy of EOC treatment. Furthermore, adipocytes are a source of various paracrine secretion products, many of which are closely related with EOC resistance to therapies.

### Adipocytes as treatment targets

As adipocytes modulate almost the whole processes of EOC progression, they could be attractive targets for EOC therapy. As mentioned above, adipocytes regulate EOC progression through diverse mechanisms, potential therapeutic targets could be proposed in various sites, steps and processes (Table 1).

**Table 1:** Potential targets and drugs against the interplay between cancer cells and adipocytes

Target	Agent	Mechanism	Anti-cancer effect	Reference
PPARγ	GW9662	Suppression of adipocyte differentiation	Inhibition of the anti-PD-L1 effect of adipocytes	[106]
adipogenesis	Sulforaphane	Suppression of adipocyte differentiation	Inhibition of the cancer promoting effect of adipocytes	[111]
β-adrenergic	Propranolol	Suppression of lypolysis in adipocytes	Inhibition of the EOC promoting effect of adipocytes	[15]
lipid droplet	Myricetin	Suppression of the lipid droplets accumulation	Inhibition of the EOC promoting effect of adipocytes	[15, 112]
β-oxidation	Trimetazidine	Inhibition of fatty acid oxidation	Inhibition of the EOC promoting effect of adipocytes	[15, 113]
CD36	Sulfo-N-succinimidyl oleate	Inhibition of fatty acid uptake of EOC cells	Inhibition of the EOC progression and metastasis induced by adipocytes	[114]
Leptin	pegylated leptin peptide receptor antagonist 2	Leptin antagonist	Inhibition of ovarian cancer peritoneal metastasis	[115]
IL-6	Tocilizumab	Monoclonal antibody against IL-6R	Enhancement of the immunity in patients with recurrent EOC	[116]
VEGF-A	Bevacizumab	Monoclonal antibody against VEGF-A	Improvement of the survival in patients with ovarian cancer	[81]

#### Suppression of adipocyte differentiation

Mature adipocytes promote the whole process of EOC progression. Therefore, suppression of adipocyte differentiation (adipogenesis), a process of the formation of adipocytes from preadipocytes or stem cells, is likely to be an effective way for EOC therapy. GW9662, a peroxisome proliferators-activated receptor (PPAR)γ antagonist, inhibited adipogenesis by suppressing nuclear receptor PPARγ, a key transcription factor promoting adipogenesis [117, 118]. GW9662 could boost the checkpoint blockade immunotherapy for distinct breast cancers [106]. Sulforaphane, a compound within the isothiocyanate group of organosulfur compound, could suppress adipocyte differentiation and decrease breast cancer formation [111]. Whether these drugs could provide anticancer effect in EOC needs to be explored in the future.

#### Suppression of the metabolic pathways

Metabolic interactions between adipocytes and EOC cells promote cancer progression. Therefore, metabolic pathways could be effective treatment targets. First, suppression lypolysis in adipocytes is suggested. Stimulation of β-adrenergic could induce lipolytic activation in adipocytes. Propranolol, the β-adrenergic receptor antagonist, partially reversed HGSOC cell–induced lipolytic activation in adipocytes [15]. Myricetin, a flavonoid found in many food sources, can suppress the lipid droplets accumulation in adipocytes [112]. Second, inhibition of fatty acid oxidation in cancer cells is also suggested. FFAs released by adipocytes are transferred to EOC for energy supply. Thus, inhibition of fatty acid oxidation in EOC cells should be able to inhibit cancer cell progression. Trimetazidine, an inhibitor of fatty acid oxidation, was reported to result a dose dependent induction of cancer apoptosis [113]. Third, inhibition the process of FFAs movement from adipocytes to EOC cells is a potential candidate. Fatty acid receptor CD36, involved in fatty acid uptake, is expressed in EOC cell surface. Blockade of CD36 decreased ovarian cancer cell metastasis [114].

#### Target therapy against adipocyte secretion products

Adipocyte secretion products modify the behavior of EOC cells, and they are the potential targets for EOC therapy. First, targeting hormones secreted by adipocytes is suggested. Leptin, a hormone secreted by adipocytes, exists in a great amount in the malignant ascites of EOC and is correlated with poor outcome of EOC patients [119, 120]. Leptin inhibition significantly suppressed ovarian malignant ascites induced metastatic aggravation of EOC cells [115]. Second, inflammatory factors secreted by adipocytes are potential targets for EOC therapy. IL-6/IL-6R, a pro-inflammatory signaling, was proposed as a therapeutic target for EOC. Tocilizumab, a monoclonal antibody of IL-6R, in combination with chemotherapy, showed a possible immunological benefit in advanced EOC patients in a phase I trial [116]. Third, angiogenic factors secreted by adipocytes are also potential targets for EOC therapy. Bevacizumab, monoclonal antibody of VEGF-A, is active in patients with advanced and recurrent ovarian cancer and improved the prognosis of ovarian cancer patients [81]. Other targets, such as exosomes secreted by adipocytes [121], are worth to be investigated for EOC precise therapy.

### Adipocytes as anticancer tools

The adipose tissue around tumor cells forms an “energy base” for cancer progression by providing nutrition, promoting tumor angiogenesis and participating inflammatory microenvironment formation. In view of this feature, it is reasonable to propose drugs added to adipocytes may be swallowed by cancer cells in the process of absorbing energy from these adipocytes. As expected, adipocytes carrying an anti-cancer fatty acid and a doxorubicin prodrug showed effective anti-cancer ability. Moreover, these adipocytes also down-regulated the PD-L1 expression level in tumor cells, favoring the emergence of CD4+ and CD8+ T cell-mediated immune responses [122]. Similarly, nanoparticle engineered tumor necrosis factor-related apoptosis-inducing ligand (TRAIL)-overexpressing adipose-derived stem cells could also work as drug-delivery vehicles for targeting and eradicating glioblastoma multiforme [123]. The potential for adipocytes in EOC therapy remains to be elucidated, especially given the important therapeutic possibilities.

### Adipocytes in treatment resistance

Tumor microenvironment is the key factor that determines the survival and drug resistance of cancer. Adipocytes, the major component of the microenvironment in EOC, have been shown to be responsible for EOC treatment resistance through a variety of mechanisms.

The main mechanism of adipocyte-induced therapy resistance is to modulate the pro-survival pathways and survival genes. It was reported adipocyte-secreted leptin contributed to the taxol chemoresistance in EOC through the activation of EMT in ovarian cancer cells [124]. Leptin could also activate AKT and ERK survival pathways in EOC cells, which are known to play crucial roles in EOC drug resistance [125]. Similarly, adipocyte-secreted cytokines, such as IL-8 and IL-6, were also reported to rapidly activated AKT or ERK survival pathways in EOC and up-regulated several genes involved in EOC survival [100, 126]. Besides, miR-21, which is abundant in the exosomes from CAAs, can produce paclitaxel resistance in EOC cells by targeting apoptotic protease activating factor 1 (APAF1) [127]. MiR-21 was also shown to promote the survival and cisplatin resistance of EOC by regulating tumor suppressor programmed cell death factor 4 (PDCD4) and cellular inhibitor of apoptosis protein 2 (c-IAP2) [128]. Together, these data indicate that adipocytes protect EOC cells via secretion of hormones, cytokines or exosomes.

Adipocytes could also protect EOC cells from chemotherapeutic agents via ECM. One of the main mechanisms of environmental protection of cancer cells from the chemotherapeutic drugs is the increase of cell adhesion to ECM [129, 130]. The drug resistance that results from direct cell contact with the ECM or other cells has been called “cell-adhesion-mediated drug resistance” or CAM-DR [131]. Adipocytes are a major source of ECM components, such as different types of collagen [132]. During tumorigenesis, cancer cells could secrete various factors that lead the neighboring adipocytes to produce ECM proteins. Indeed, adipocytes in close contact with cancer cells could secrete high levels of collagen VI [133]. EOC cells adhered to collagen VI exhibited an increase survival when exposed to cisplatin, possibly through up-regulating metallothioneins which play important roles in cisplatin resistance [134].

Adipocyte-induced autophagy activation may also play important roles in drug resistance of EOC. The effect of autophagy on cancer is complex, which is illustrated by the identification that autophagy plays a double sword role in cell death and cell survival in cancer. Autophagy is considered to play an inhibitory role in tumor initiation. After tumor formation, autophagy always plays a positive role in malignant progression and drug resistance [135]. Autophagy could be induced in response to EOC chemotherapy, and was shown to cause chemotherapy resistance [136, 137]. Mammalian target of rapamycin (mTOR) is a central regulator of autophagy [135]. AKT/mTOR-mediated autophagy could induce drug resistance in ovarian cancer [138]. Moreover, the chemosensitivity of ovarian cancer could be enhanced by suppressing autophagy via mTOR pathway activation [139]. It was reported adipocytes protected the myeloma cells from chemotherapy-induced apoptosis by activating autophagy and up-regulating autophagic protein expression. Adipocyte-secreted adipokines, such as leptin and adipsin, were responsible for the adipocyte-induced autophagy and therapy resistance of myeloma [140]. However, the roles of adipocytes in regulating autophagy in EOC remain to be elucidated, especially considering the important therapeutic possibilities.

Adipose tissue contains abundant mensenchymal stem cells. These adipose-derived stem cells (ASCs) play important roles in the promotion of EOC chemoresistance. ASCs derived from the human omentum increase the paclitaxel or carboplatin resistance of EOC cells [65]. Importantly, it is reported that ASCs induced the chemoresistance of EOC partly through regulating nitric oxide pathway [141]. Moreover, ASCs could enhance autophagy in EOC cells [142], which was reported to cause chemoresistance in EOC [136, 137]. Future studies are needed to investigate the impact of targeting ASCs on EOC chemoresistance. Furthermore, the detailed analysis of altered molecular pathways of EOC cells in the presence of ASCs will allow the development of targeted therapies.

## Conclusions

Given the striking association between obesity and cancer, adipocytes have attracted more and more attention from researchers. Growing evidence has transformed adipocytes from silent bystanders into active facilitators in cancer progression. Adipocytes, close neighbors of EOC and major components of EOC microenvironment, are involved in almost all EOC progression processes and may become potential targets for EOC treatment. Considering the important EOC promoting effect of adipocytes, in depth mechanism regarding the influence of adipocytes on EOC biology needs to be elucidated. Moreover, it is important to explore the crosstalk between adipocytes and EOC, which leads to the modification of adipocyte phenotype and biological behavior. Furthermore, because high fat diet, the main cause of obesity, can affect the initiation and progression of EOC [143, 144], we propose that EOC patients, especially those who are obese, may benefit from special life style interventions, such as diet and exercise. Whether life style interventions are sufficient to improve the prognosis of EOC needs to be elucidated in the future.

## Data Availability

Not applicable.

## Abbreviations

EOC: Epithelial ovarian cancer
TNFα: Tumor necrosis factor α
IL: Interleukin
17βHSD: 17β-hydroxysteroid dehydrogenase
ATME: Adipose tissue microenvironment
HGSOC: High-grade serous ovarian cancer
CAAs: Cancer associated adipocytes
EMT: Epithelial mesenchymal transition
ZEB1: Zinc finger E-box binding homeobox 1
STAT3: Signal transducer and activator of transcription 3
ERK: Extracellular regulated protein kinases
VEGF: Vascular endothelial growth factor
ECM: Extracellular matrix
IGF-1: Insulin-like growth factor 1
AKT: Serine/threonine protein kinase
MCP-1: Monocyte chemoattractant protein-1
TIMP: Tissue inhibitors of matrix metalloproteinases
CD: Cluster of differentiation
JNK: c-Jun N-terminal kinase
HGF: Hepatocyte growth factor
MMP: Matrix metallopeptidase
FFAs: Free fatty acids
BCL-2: B-cell lymphoma 2
BCL-X_L_: B-cell lymphoma-extra large
BAX: BCL2-associated X protein
Bim: Bcl-2 interacting mediator of cell death
AMPK: AMP-activated protein kinase
CPT-1: Carnitine palmitoyltransterase 1
CAFs: Cancer-associated fibroblasts
HSL: Hormone-sensitive lipase
HIF: Hypoxia inducible factor
CLSs: Crown-like structures
BMI: Body mass index
ATM: Adipose tissue macrophage
NF-κB: Nuclear factor kappa B
PD-L1: programmed death-ligand 1
PD-1: Programmed cell death protein 1
IFN: Interferin
PPAR: Peroxisome proliferators-activated receptor
TRAIL: Tumor necrosis factor-related apoptosis-inducing ligand
APAF1: Apoptotic protease activating factor 1
PDCD4: Programmed cell death factor 4
c-IAP2: Cellular inhibitor of apoptosis protein 2
CAM-DR: Cell-adhesion-mediated drug resistance
mTOR: Mammalian target of rapamycin
ASCs: Adipose-derived stem cells
